# Frovatriptan vs. other triptans for the acute treatment of oral contraceptive-induced menstrual migraine: pooled analysis of three double-blind, randomized, crossover, multicenter studies

**DOI:** 10.1007/s10072-013-1393-x

**Published:** 2013-05-22

**Authors:** G. Allais, V. Tullo, S. Omboni, D. Pezzola, D. Zava, C. Benedetto, G. Bussone

**Affiliations:** 1 Department of Gynecology and Obstetrics, Women’s Headache Center, University of Turin, Via Ventimiglia 3, 10126 Turin, Italy; 2 Department of Clinical Neuroscience, National Neurological Institute Carlo Besta, Milan, Italy; 3 Clinical Research Unit, Italian Institute of Telemedicine, Varese, Italy; 4 Medical Department, Istituto Lusofarmaco D’Italia, Milan, Italy

**Keywords:** Almotriptan, Estrogen withdrawal, Frovatriptan, Oral-contraceptive induced menstrual migraine, Rizatriptan, Zolmitriptan

## Abstract

Oral contraceptive-induced menstrual migraine (OCMM) is a particularly severe form of migraine triggered by the cyclic hormone withdrawal. To review the efficacy of frovatriptan vs. other triptans, in the acute treatment of OCMM through a pooled analysis of three individual randomized Italian studies. With or without aura migraineurs were randomized to frovatriptan 2.5 mg or rizatriptan 10 mg (study 1), frovatriptan 2.5 mg or zolmitriptan 2.5 mg (study 2), frovatriptan 2.5 mg or almotriptan 12.5 mg (study 3). All studies had a multicenter, randomized, double-blind, crossover design. After treating 1–3 episodes of migraine in 3 months with the first treatment, patients switched to the other treatment for the next 3 months. In this analysis, the subset of 35 of the 280 women of the intention-to-treat population taking combined oral contraceptives and experiencing a migraine attack during the withdrawal phase, were analyzed. The proportion of pain free and pain relief at 2 h were 25 and 51 % with frovatriptan and 28 and 48 % with comparators (*p* = NS). At 24 h, 71 and 83 % of frovatriptan-treated patients and 60 and 76 % of comparator-treated patients were pain free (*p* < 0.05 between treatments) and had pain relief (*p* = NS), respectively. Relapse at 24 and 48 h was significantly (*p* < 0.05) lower with frovatriptan (17 and 21 %) than with the comparators (27 and 31 %). Our results suggest that, due to its sustained antimigraine effect, frovatriptan may be particularly suitable for the management of OCMM than other triptans.

## Introduction

Oral contraceptive-induced menstrual migraine (OCMM) is a menstrual migraine subtype mainly caused by estrogen withdrawal occurring during the week of pill suspension [1, 2]. OCMM is a poorly studied, but particularly severe and disabling form of menstrual migraine [3]; it is codified by the ICHDII classification as estrogen-withdrawal headache (code 8.4.3) [2].

Evidence-based guidelines recommend treating moderate or severe migraine, including menstrual migraine, with migraine-specific agents [4]. Among these, triptans are considered to provide a greater clinical benefit and a more favorable adverse event profile than other anti-migraine drugs [4].

Frovatriptan is a second-generation triptan, with a distinct pharmacokinetic profile. Its high selectivity for the cerebral vasculature, long elimination half life and high persistence of therapeutic action may be useful in preventing menstrual migraine, but also for treating acute attacks, as shown by several randomized trials [4–7]. Although frovatriptan is currently among the triptans recommended for prevention of menstrual migraine [8–10], its efficacy in OCMM has had limited investigation [11]. In the present retrospective analysis, we report on a pooled individual data analysis of three randomized double-blind studies [12–14] performed to verify whether frovatriptan may be effective in the acute management of an OCMM attack and whether its efficacy may differ from that of other triptans.

## Methods

### Study population and design

Information on the study design for the three original studies is reported in the main publications [12–14]. In brief, the studies recruited subjects of both genders, aged 18–65 years, with a current history of migraine with or without aura, according to the International Headache Society definition [2], and with at least one, but no more than six migraine attacks per month for 6 months prior to entering the study. In the present retrospective analysis, only the group of women with OCMM was taken into consideration. OCMM was defined according to IHS criteria, as an estrogen-withdrawal headache, namely a migraine without aura developing within 5 days after cessation of estrogen use and resolving within 3 days [2]. Estrogen use must have lasted for at least 3 weeks prior to cessation. Patients taking all types of combined oral contraceptives, except those entailing a withdrawal period of less than 5 days, were considered eligible for inclusion in the present retrospective analysis.

The studies had a multicenter, randomized, double blind, cross-over design. A total of 33 different Italian centers were involved in the studies. Each patient received frovatriptan 2.5 mg or rizatriptan 10 mg in the first study [12], frovatriptan 2.5 mg or zolmitriptan 2.5 mg in the second study [13], and frovatriptan 2.5 mg or almotriptan 12.5 mg in the third study [14], in a randomized sequence. After treating 1–3 episodes of migraine in no more than 3 months with the first treatment, the patient switched to the other treatment and was asked to treat a maximum of three episodes of migraine in no more than 3 months with the second treatment.

The study design foresaw three visits and each patient’s participation time in the study had not to exceed 6 months from randomization. Subjects having no migraine episodes during one of the two observation periods were excluded from the study.

Randomization was done by blocks of four. Blindness ensured by the overencapsulation technique, i.e., by inserting study drug tablets in capsules.

### Data analysis

The present pooled analysis was carried out in all women with OCMM, who treated at least one episode of OCMM with both medications in each study.

The following endpoints were evaluated: (a) the proportion of pain-free episodes at 2 and 24 h (absence of migraine episodes 2 and 24 h after intake of one dose of study drug); (b) the proportion of pain-relief episodes at 2 and 24 h (a decrease in migraine intensity from severe or moderate to mild or none 2 and 24 h after the intake of one study drug dose); (c) relapse within 24 and 48 h (defined as an episode of migraine occurring within 24 and 48 h from the previous episode, after a period without migraine).

Continuous variables were summarized by computing average values and standard deviations (SD), while categorical variables by computing the absolute value and the frequency (as percentage). Study endpoints were compared between groups by a Student’s *t* test (continuous variables) or by a *χ* ^2^ test (categorical variables). The level of statistical significance was kept at 0.05 for all analyses.

## Results

**Fig. 1:**
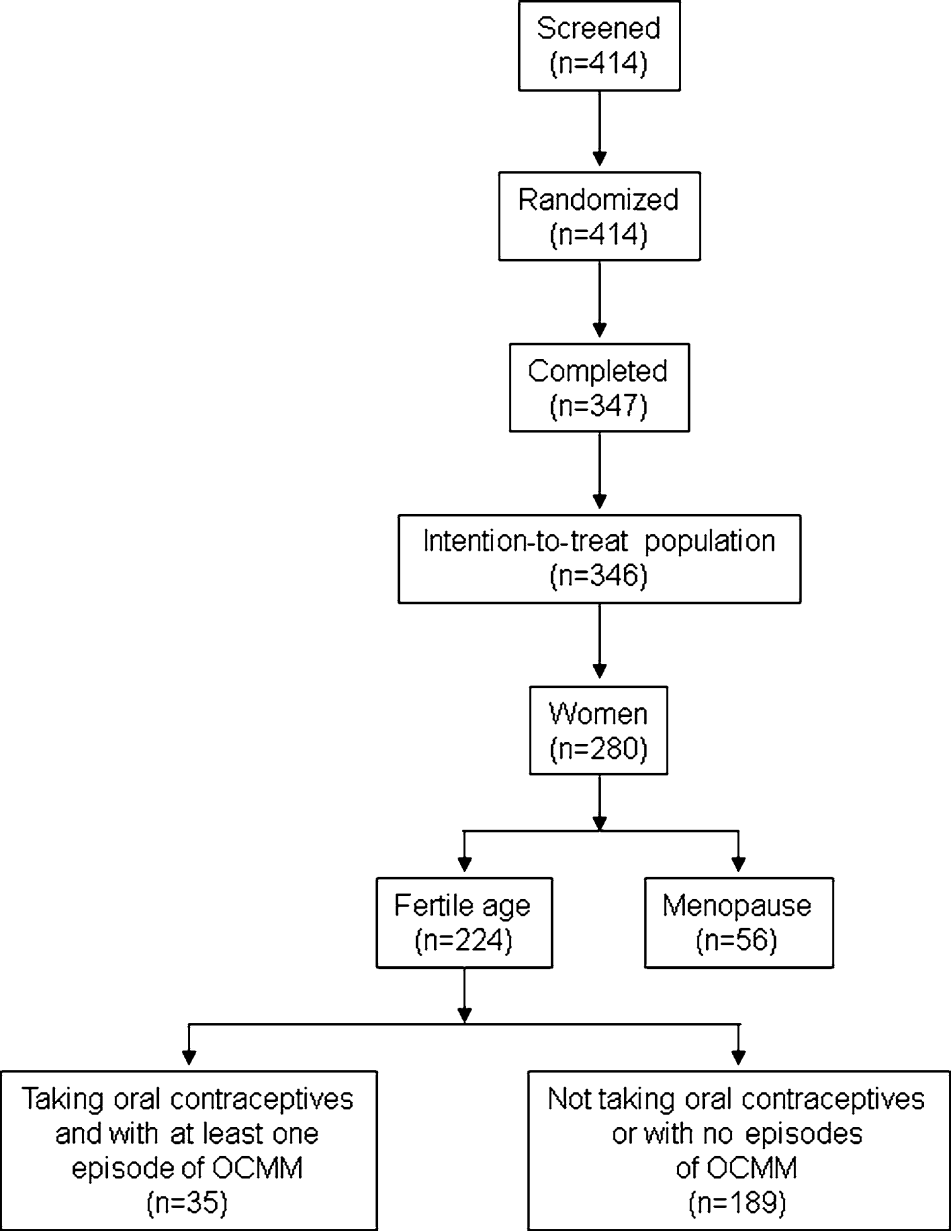
Flow diagram of the patients throughout the study

The main study population consisted of 346 subjects, of which 280 (81 %) were women and 224 were in the fertile age. A total of 35 out of the 224 eligible women (16 %) were taking oral contraceptives, treated at least one episode of OCMM with both medications and were thus included in the present analysis. A flow diagram of participants throughout the study is summarized in Fig. 1.

Demographic and clinical baseline data of the 35 women with OCMM attacks and of the 189 women with non-OCMM attacks are reported in Table 1. No statistically significant differences were observed between the two subgroups of women for any of the explored variables.

**Table 1:** Baseline demographic and clinical data of the 35 women with oral contraceptive-induced menstrual migraine (OCMM) and of the 189 women with non-OCMM of the intention-to-treat population

	Women with OCMM (*n* = 35)	Women with non-OCMM (*n* = 189)	*p* value
Age (years, mean ± SD)	35 ± 9	34 ± 7	NS
Height (cm, mean ± SD)	163 ± 5	164 ± 6	NS
Weight (kg, mean ± SD)	59 ± 10	59 ± 10	NS
Age at onset of migraine (years, mean ± SD)	18 ± 7	16 ± 6	NS
Migraine attack duration >2 days (*n*, %)	7 (20)	45 (24)	NS
MIDAS score (mean ± SD)	22 ± 20	23 ± 15	NS
No use of triptans in the previous 3 months (*n*, %)	16 (46)	79 (42)	NS
Migraine attacks with aura (*n*, %)	4 (10)	29 (10)	NS

Data are shown as mean (±SD), or absolute (*n*) and relative frequency (%). *p* value refers to the statistical significance of the between-group difference

A total of 144 out of the overall 1,289 attacks occurring in the 224 women in fertile age were classified as OCMM: 73 of the 144 attacks (51 %) were treated with frovatriptan and 71 (49 %) with comparators (8 women treated with rizatriptan, 12 with zolmitriptan and 15 with almotriptan).

**Fig. 2:**
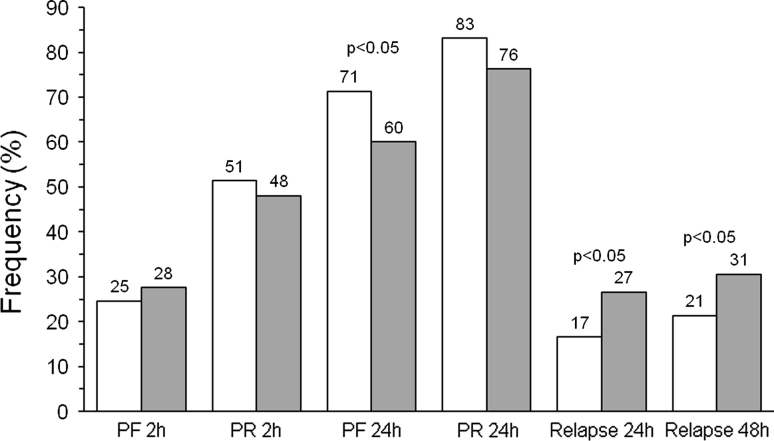
Main study endpoints in the group of women with oral contraceptive-induced menstrual migraine (OCMM) treated with frovatriptan (*open bars*) and the comparators (*grey bars*). Data are reported as relative (%) frequencies for pain free (PF) at 2 and 24 h, for pain relief (PR) at 2 and 24 h, and for relapses at 24 and 48 h. The *p* values on *top of the bars* refer to the level of the statistical significance of the difference between the two study drugs

As shown in Fig. 2, the proportion of pain free and pain-relief episodes at 2 h were not significantly (*p* = NS) different between frovatriptan (25 and 51 %) and the comparators (28 and 48 %, respectively). At 24 h, a larger proportion of women treated with frovatriptan were pain free (71 vs. 60 % comparators, *p* < 0.05) and had pain relief (83 vs. 76 % comparators, *p* = NS).

Finally, the rate of relapses at 24 and 48 h was significantly (*p* < 0.05) lower with frovatriptan (17 and 21 %) than with the comparators (27 and 31 %) (Fig. 2).

## Discussion

As shown in numerous randomized trials, frovatriptan represents a well known and effective option for acute [4–6, 11] or prophylactic treatment [15–17] of menstrual migraine. The present study, based on a pooled analysis of three double-blind, randomized, direct comparative, cross-over studies [12–14], demonstrates that frovatriptan is clinically effective also for the acute treatment of OCMM, a particular menstrual migraine subtype.

According to our results, the clinical efficacy of frovatriptan at 2 h was similar to that of the other triptans, with the overall pain-free rates of approximately 25 % and of pain-relief rates of approximately 50 %. As expected, such proportions progressively increased during the period of observation, reaching larger values at 24 h under frovatriptan. In particular, this drug showed a more sustained relieving effect on migraine than the other triptans, with lower headache relapses after 24 h and even more so after 48 h and such findings might be explained by differences in the pharmacokinetics of frovatriptan when compared with the other triptans [7, 18].

Very poor evidence is available on the efficacy of triptans in OCMM and no previous direct comparison studies of various triptans have ever been published. In a pilot, open label, uncontrolled study, 20 women treating an OCMM acute attack with frovatriptan 2.5 mg displayed pain-free and pain-relief rates of 10 and 55 % at 2 h, and of 60 and 75 % at 24 h [11]. Migraine relapsed within 24 h in 36 % of patients [11]. In a cross-over, randomized study, 46 % of the women taking 50 mg of oral sumatriptan achieved pain relief at 2 h and 18 % were pain free, with almost comparable results observed with the suppository formulation of sumatriptan [19].

An additional interesting finding of our retrospective analysis is that immediate and delayed efficacy of frovatriptan in the subgroup of OCMM is superimposable to that previously observed in the whole subgroup of women with menstrual migraine [6].

Despite the interesting results, we must recognize the limitation of the post hoc nature of our analysis. However, to our knowledge, no comparative prospective studies of triptans in OCMM are yet available or have been planned. Our results might be helpful to stimulate the design and implementation of larger direct comparative randomized clinical trials evaluating triptan efficacy in such a specific subtype of menstrual migraine.

In conclusion, our analysis of individual data of double-blind, randomized, cross-over trials, suggests that frovatriptan and other widely employed triptans share a similar efficacy in the immediate treatment of acute attack of OCMM. However, frovatriptan due to its sustained antimigraine effect, may be more suitable for the management of OCMM than other triptans.
